# Ruptured Intracranial Mycotic Aneurysm in Infective Endocarditis: Multidisciplinary Management to Determine Timing of Cardiac Surgery

**DOI:** 10.7759/cureus.115323

**Published:** 2026-08-27

**Authors:** Sahibjot Bhatia, Joel Varughese, Navjiwan Bhandal, Bertrand Liang

**Affiliations:** 1 Neurology, St. Joseph's Medical Center, Stockton, USA; 2 Internal Medicine, St. Joseph's Medical Center, Stockton, USA; 3 Department of Neurology, University of Colorado School of Medicine, Colorado Springs, USA

**Keywords:** cerebral mycotic aneurysm, cerebrovascular complications, enterococcus faecalis (e. faecalis), infective endocarditis, intracranial hemorrhage, ischemic stroke, neurosurgical clipping, septic embolism, subarachnoid hemorrhage

## Abstract

Infective endocarditis (IE) is a disease process associated with significant morbidity and mortality due to systemic embolization and metastatic infection. We present the case of a 65-year-old male with an extensive past medical history who presented with chest pain and dyspnea and was found to have *Enterococcus faecalis* aortic valve IE complicated by severe aortic regurgitation. His complex hospital course included non-ST-elevation myocardial infarction (NSTEMI) secondary to septic coronary embolization, as well as cerebrovascular embolization causing a left parieto-occipital infarct. The coronary embolization was favored to be septic in etiology given the presence of an aortic valve vegetation, concurrent *Enterococcus faecalis* bacteremia, and the absence of an alternative explanation for the acute distal coronary occlusion. Neurovascular evaluation and digital subtraction angiography confirmed a distal intracranial mycotic aneurysm causing hemorrhage. The complexity of this case necessitated extensive multidisciplinary collaboration among neurology, neurosurgery, cardiothoracic surgery, infectious disease, neurointerventional radiology, and other specialty services to find the source of infection and balance the risks of intracranial hemorrhage, ongoing infection, and urgent valve replacement. This case highlights how neurological complications can dictate the sequence and timing of cardiac surgery to achieve favorable outcomes in patients with complex disseminated* Enterococcus faecalis* IE.

## Introduction

Infective endocarditis (IE) is a rare and potentially life-threatening disease characterized by the colonization of virulent organisms on the cardiac valve endocardium [1]. Despite advanced diagnostics, antimicrobial therapy, and surgical management, IE is a condition associated with significant morbidity and mortality due to its potential complications [1,2]. Systemic embolization is a known complication that occurs in approximately 20-40% of individuals with IE [3]. Cerebral infarctions, cerebral hemorrhages, and rarely hemorrhagic transformation of cerebral infarctions can be complications secondary to these systemic embolizations [3]. Acute coronary syndrome secondary to septic emboli is known to be a rare complication with overall poor outcomes [4]. *Enterococcus faecalis* is a known cause of IE, typically seen in individuals who may have urinary tract infections, abdominal infections, aortic valve disease, and colorectal cancers [5]. Although there are multiple variations of complications that can be seen in IE, there are also reported cases of mycotic aneurysms that introduce distinct neurovascular risks [6]. Recent literature highlights the substantial morbidity associated with ruptured intracranial mycotic aneurysms and the lack of standardized management strategies due to their rarity [7]. In this report, we present a complex case of an *Enterococcus faecalis* aortic valve IE complicated by septic coronary artery embolization causing non-ST-elevation myocardial infarction (NSTEMI), ischemic stroke with intracranial hemorrhage secondary to a ruptured mycotic aneurysm, and metastatic vertebral discitis/osteomyelitis requiring staged neurosurgical intervention prior to definitive valve replacement. Although neurologic complications of IE are well described in the literature, management becomes particularly challenging when a ruptured intracranial mycotic aneurysm occurs in a patient who simultaneously has a surgical indication for severe aortic regurgitation. The sequence and timing of neurosurgical and cardiac interventions in this setting remain individualized due to the need for anticoagulation during cardiopulmonary bypass, which may increase the risk of or worsen ongoing intracranial hemorrhage. This case presents a staged, multidisciplinary approach that highlights how neurologic complications can dictate the sequence and timing of definitive cardiac surgery in IE.

## Case presentation

A 65-year-old male with end-stage renal disease (ESRD) on hemodialysis, hypertension, type 2 diabetes mellitus, atrial fibrillation on apixaban, schizophrenia, heart failure with preserved ejection fraction (HFpEF), and prostate cancer presented with two days of progressive shortness of breath and one day of pressure-like chest pain radiating to the left arm. 

On arrival, vital signs showed a temperature of 36.7°C, heart rate of 97 beats/min, respiratory rate of 38 breaths/min, blood pressure of 118/65 mmHg, and improved oxygenation on continuous positive airway pressure (CPAP). Neurologic examination was unremarkable. Initial laboratory evaluation is summarized in Table 1. He was treated with an unfractionated heparin infusion, initiated with a 4,000-unit bolus (60 units/kg; maximum 4,000 units), followed by an infusion at 10.65 units/kg/hr for presumed NSTEMI in the setting of markedly elevated troponin level, with subsequent adjustments based on anti-Xa levels. Transthoracic and transesophageal echocardiograms demonstrated a large aortic valve vegetation with severe aortic regurgitation and possibly a small mitral valve vegetation. Blood cultures grew *Enterococcus faecalis*, and combination therapy with ampicillin 2,000 mg IV every 12 hours, ceftriaxone 2,000 mg IV every 12 hours, and gentamicin 80 mg IV with dosing per pharmacy protocol was started. The patient ultimately underwent coronary angiography, which showed distal left anterior descending (LAD) artery occlusion, believed to represent septic coronary embolization. 

**Table 1:** Initial laboratory findings

Laboratory	Result	Normal
White blood cell count	12.6 thousand/uL	4.0-10.0 thousand/uL
Hemoglobin	7.0 g/dL	13.6-17.0 g/dL
Blood urea nitrogen	39.8 mg/dL	8.4-25.7 mg/dL
Creatinine	3.2 mg/dL	0.7-1.3 mg/dL
Alanine aminotransferase (ALT)	477 Units/L	0-55 Units/L
Aspartate aminotransferase (AST)	631 Units/L	5-34 Units/L
Lactic acid	2.5 mmol/L	0.5-2.0 mmol/L
Troponin	10.431 ng/mL	0-0.033 ng/mL
Blood cultures	Enterococcus faecalis	No growth

**Figure 1:**
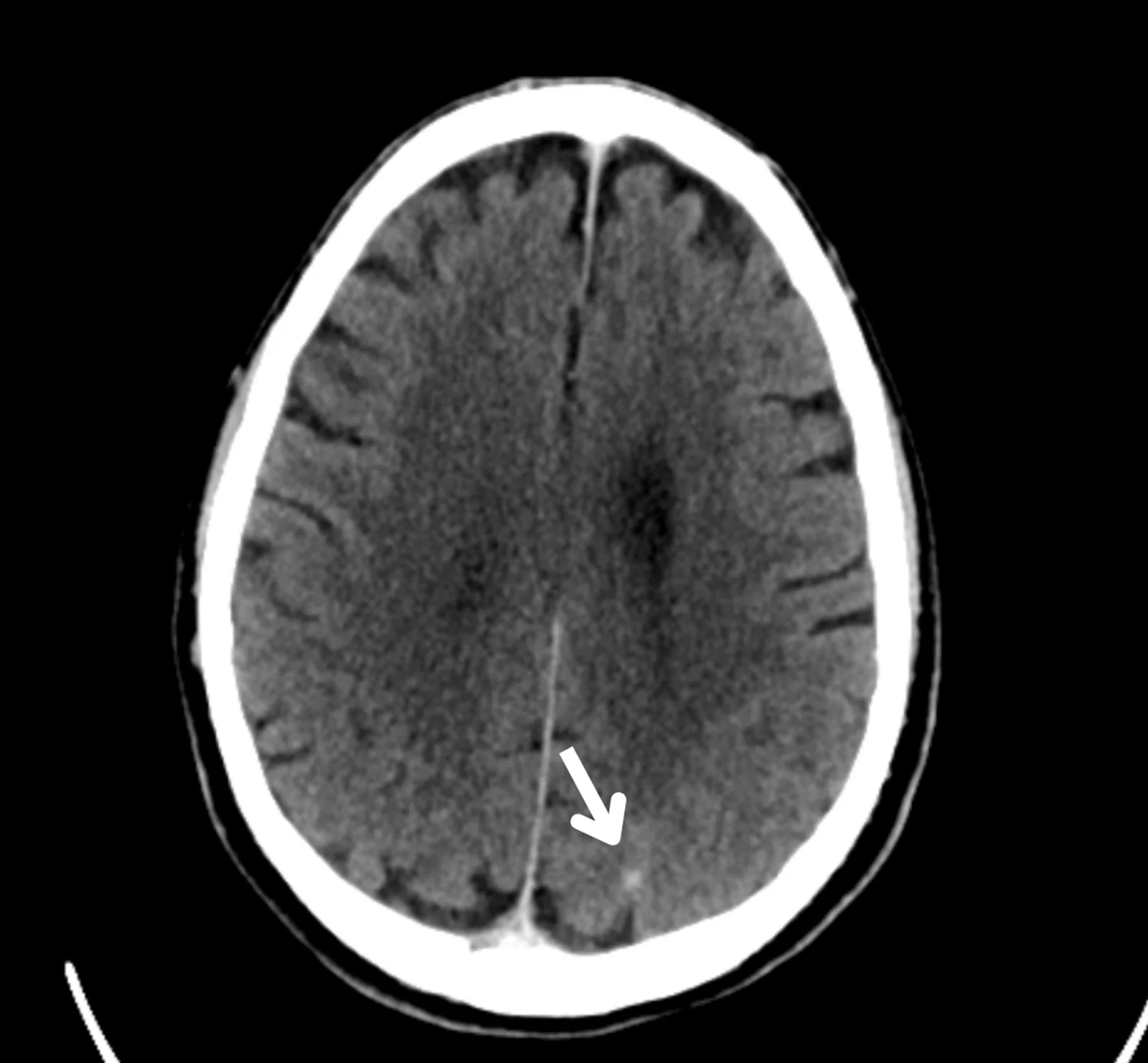
Computed tomography (CT) head with contrast Arrow showing a subtle focal hyperdensity along the left parietal extra-axial space, concerning for intracranial hemorrhage, which prompted further neurovascular evaluation

**Figure 2:**
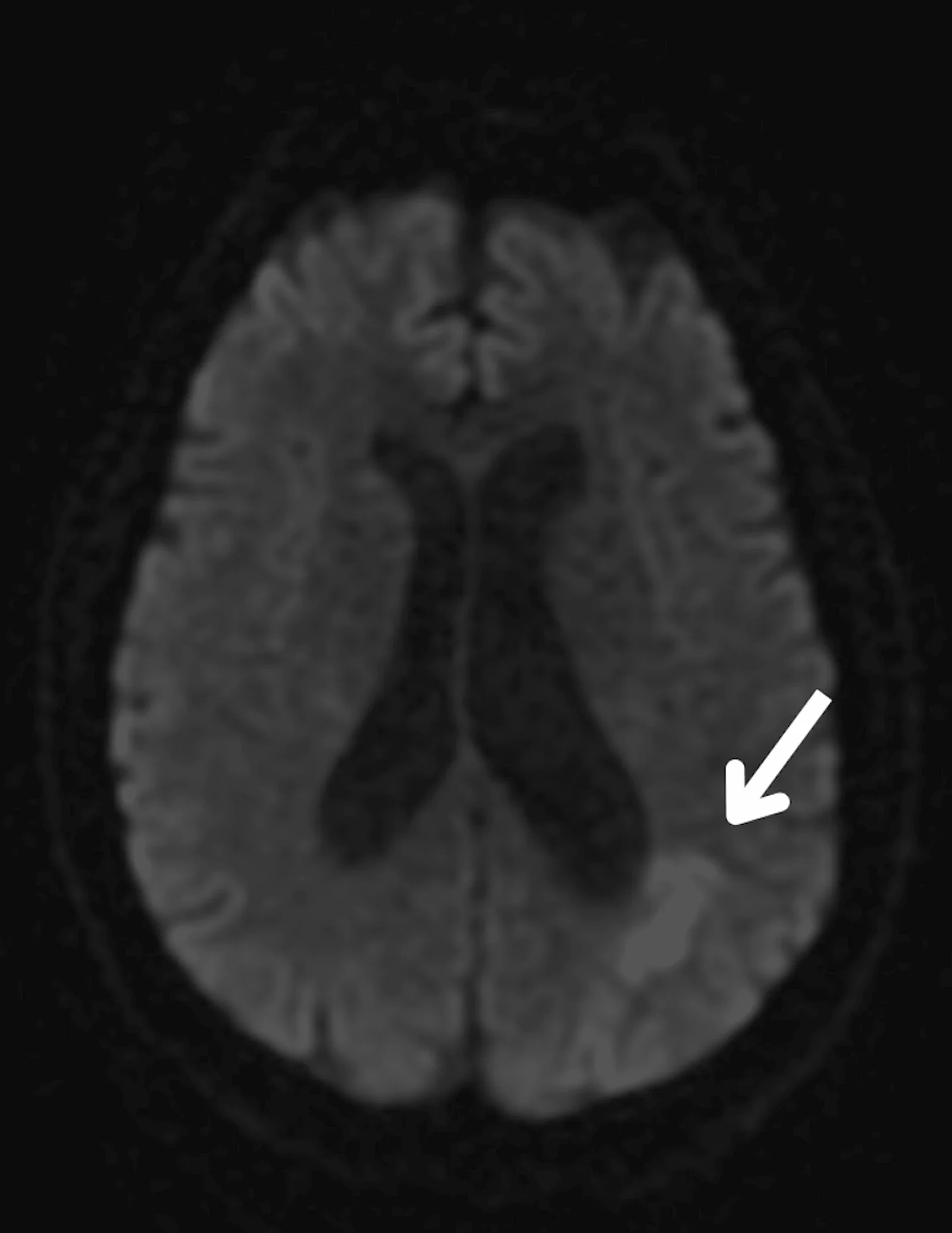
Magnetic resonance imaging (MRI) brain without contrast, diffusion-weighted imaging (DWI) sequence Arrow indicating an area of diffusion restriction in the left parieto-occipital white matter, consistent with an acute/subacute infarction in the setting of septic embolization

**Figure 3:**
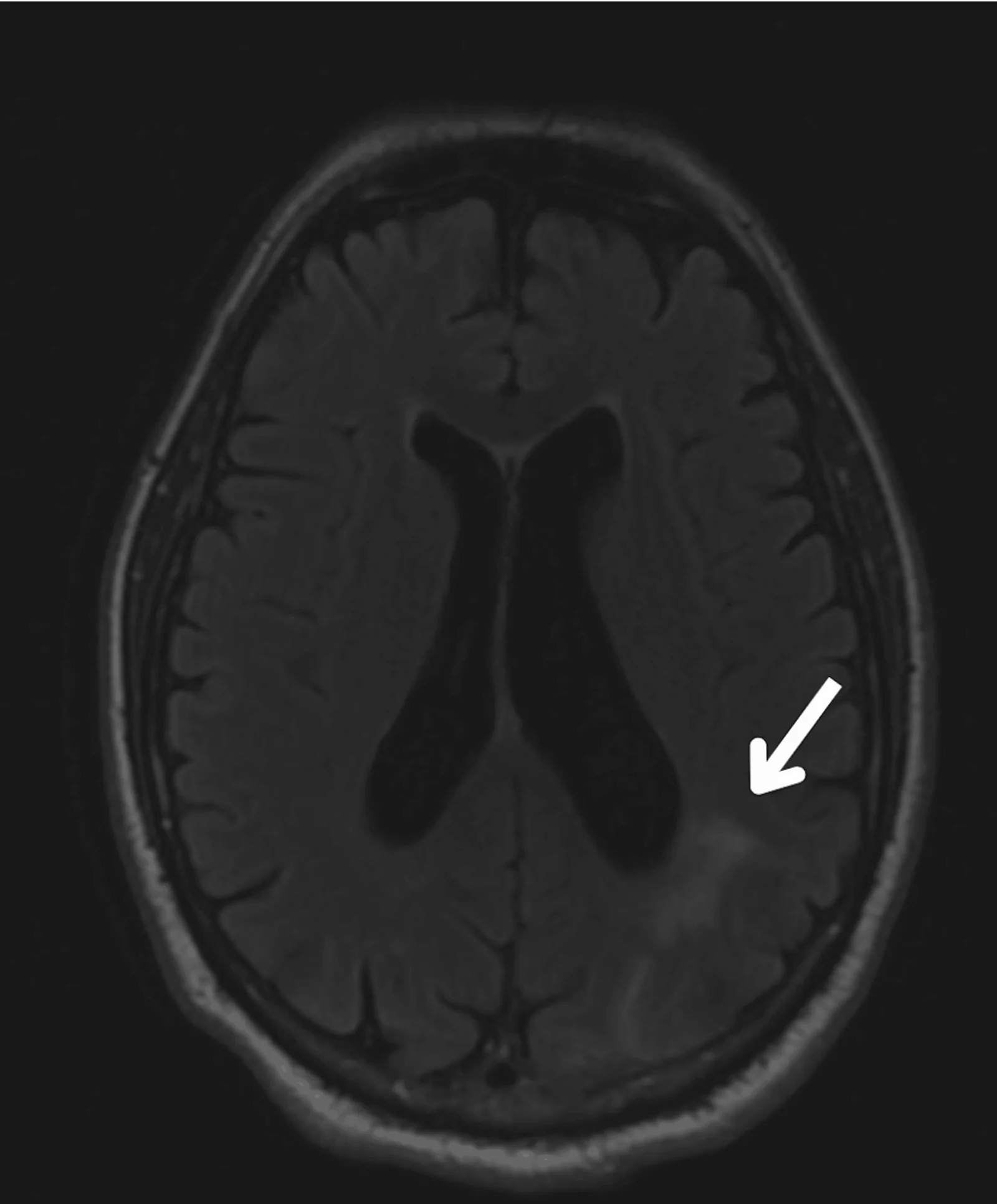
Magnetic resonance imaging (MRI) brain without contrast, fluid-attenuated inversion recovery (FLAIR) sequence Arrow demonstrating hyperintensity in the left parieto-occipital white matter, supporting the diagnosis of infarction

During hospitalization, the patient developed altered mentation and lethargy without focal neurologic deficits. Computed tomography (CT) of the head with contrast (Figure 1) demonstrated a small left parietal extra-axial hyperdensity concerning for subarachnoid versus subdural hemorrhage, with interval enlargement on repeat imaging later that day. Magnetic resonance imaging (MRI) of the brain without contrast (Figures 2, 3) subsequently demonstrated a subacute left parieto-occipital infarct with stable small-volume subarachnoid hemorrhage (SAH). Anticoagulation and aspirin were discontinued. Computed tomography angiography (CTA) of the head and neck with contrast was initially interpreted as negative; however, neurointerventional review identified a distal mycotic aneurysm that was not amenable to endovascular intervention. Digital subtraction angiography (DSA) confirmed a 2 mm pseudoaneurysm of the left posterior parietal branch of the left middle cerebral artery. MRI of the thoracic and lumbar spine with and without contrast demonstrated enhancement at T7-T8 and L2-L3, concerning for early osteomyelitis/discitis, presumed to be secondary to hematogenous spread of infection.

Evaluation for a gastrointestinal source of infection with colonoscopy was deferred in the setting of intracranial hemorrhage. Thoracolumbar discitis/osteomyelitis was managed medically, and a periodontally compromised tooth was extracted as a potential infectious source.

Given the risk of intracranial hemorrhage with systemic anticoagulation required for cardiopulmonary bypass, neurosurgery recommended surgical treatment of the mycotic aneurysm prior to valve replacement. The patient underwent left craniotomy with clipping of the mycotic aneurysm. 

Valve replacement was delayed based on neurosurgery recommendations after the development of a postoperative subdural hematoma with mild midline shift, which progressed on serial CT imaging; therefore, the patient underwent middle meningeal artery (MMA) embolization by neurointerventional radiology. Follow-up imaging demonstrated interval improvement and stabilization of the hemorrhage, after which neurology and neurosurgery cleared the patient for anticoagulation and eventual cardiac surgery. The patient underwent successful bioprosthetic aortic valve replacement (AVR). He was eventually restarted on apixaban 5mg twice daily prior to discharge after neurology and neurosurgery clearance.

The patient completed prolonged ampicillin and ceftriaxone therapy per infectious disease recommendations. Antibiotics were continued after blood cultures had cleared due to the extensive metastatic infectious complications and timing of valve replacement.

**Figure 4:**
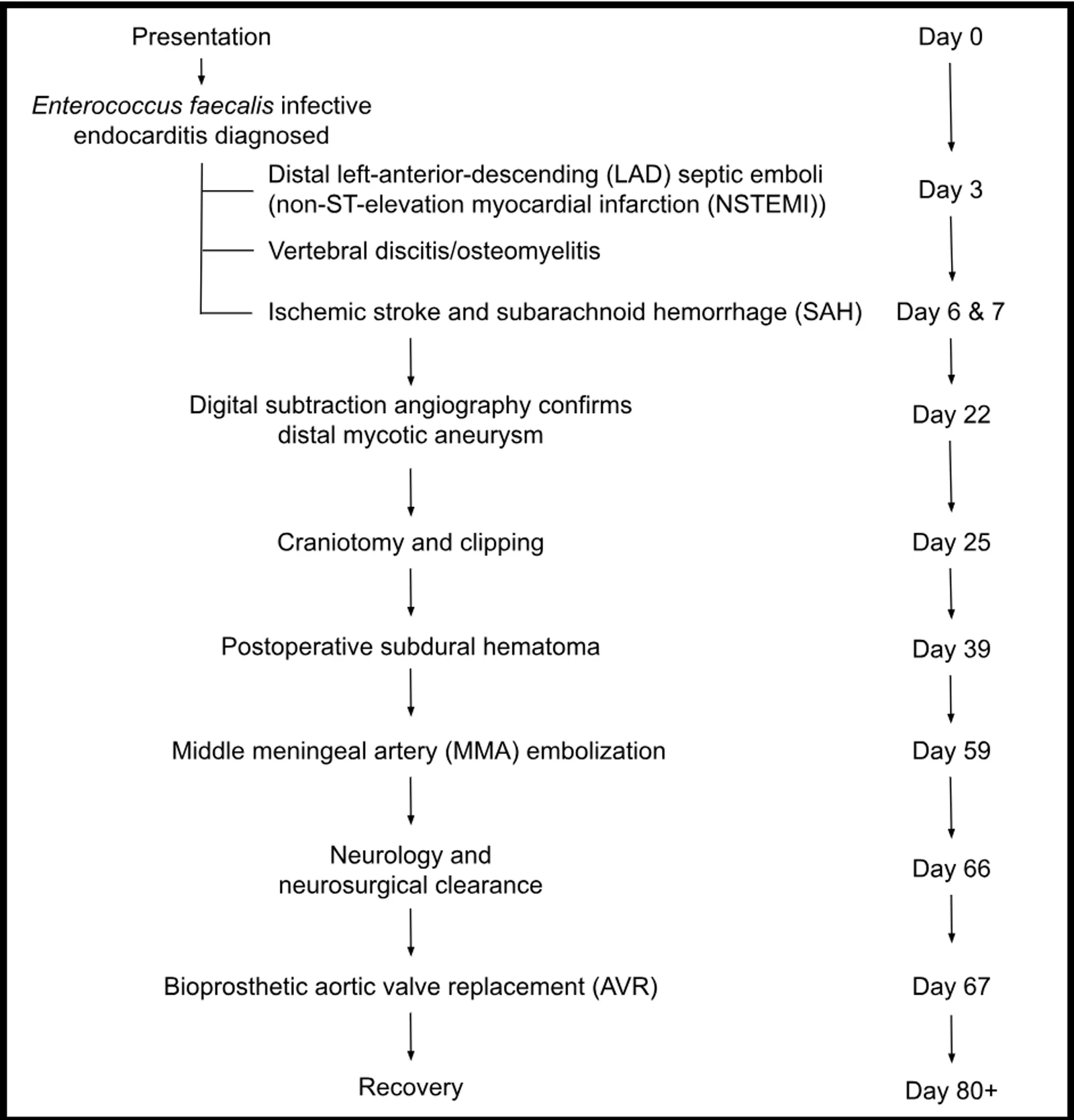
Chronological timeline of major diagnostic and therapeutic events during hospitalization

At follow-up, he was noted to be recovering well without major postoperative complications. Figure 4 shows a brief summary and chronological order of events. 

## Discussion

IE is a potentially devastating disease process that is associated with high morbidity and mortality despite substantial advances in antimicrobial and surgical management options. This case highlights an extensive pattern of embolic dissemination from Enterococcus faecalis endocarditis, resulting in coronary artery embolism, ischemic stroke, and intracranial mycotic aneurysm with SAH. Recognition of the varying sequelae of IE is critical, as they will likely alter management strategies, particularly regarding anticoagulation and surgical intervention. 

Neurologic sequelae from IE remain among the most common non-cardiac complications and also contribute to high morbidity and mortality rates [8]. Intracranial mycotic aneurysms, which arise from septic embolization resulting in focal vessel destruction, can not only significantly alter patient outcomes but also change the treatment options and timeline. Diagnosis of mycotic aneurysm is approached using imaging modalities, which include contrast CT or MRI studies, with digital subtraction angiography as a more invasive option. An important feature of this case was the diagnostic challenge in identifying the mycotic aneurysm. The initial CTA did not clearly identify the vascular lesion on radiologist review; however, due to the concern for ongoing septic embolic disease, subsequent neurovascular evaluation and digital subtraction angiography confirmed a distal intracranial mycotic aneurysm. This finding highlights the limitations of noninvasive vascular imaging for small distal aneurysms and reinforces the role of catheter angiography when clinical suspicion remains high despite nondiagnostic CTA findings.

Management of intracranial mycotic aneurysms can vary with factors, such as aneurysm location, rupture status, and neurologic stability influencing treatment selection. Proposed management varies from prolonged intravenous (IV) antibiotic therapy in the setting of unruptured aneurysms to endovascular and surgical approaches [6]. In this patient, the aneurysm was considered unsuitable for endovascular treatment, and it was instead treated with microsurgical clipping. The decision was also influenced by the anticipated need for aortic valve replacement and systemic anticoagulation. 

Management of ruptured mycotic aneurysms in IE is not restricted to a single modality. A national prospective cohort analysis of mycotic aneurysms in IE found that ruptured mycotic aneurysms, whether intracranial or extracranial, were managed with either endovascular treatment or open surgery, with the approach guided by aneurysm location, size, mass effect, and surgical risk [9]. Where anatomically feasible, endovascular treatment has been favored in recent case experience, largely because its minimally invasive nature may allow earlier initiation of anticoagulation for cardiopulmonary bypass, potentially expediting cardiac surgery; open surgical treatment has correspondingly been reserved for ruptured aneurysms with significant mass effect or those unsuitable for or unresponsive to transcatheter approaches [10]. In our patient, the aneurysm's distal location and small caliber (2 mm) were judged by the neurointerventional team to be anatomically inaccessible for endovascular treatment, leaving microsurgical clipping as the only available means of securing the aneurysm before cardiopulmonary bypass anticoagulation could be considered. 

This case also highlights that management priorities in IE are fluid and can shift as new complications arise. Although a multidisciplinary team was involved in this case, the central challenge was appropriately evaluating and proceeding with diagnostic and therapeutic interventions when multiple disease processes create competing management priorities. 

Severe aortic regurgitation from IE is an established indication for surgical management with early valve replacement [11]. In this patient, however, recent intracranial hemorrhage and an untreated mycotic aneurysm made immediate surgery risky. Neurologic stabilization became the immediate priority, which delayed cardiac intervention. The 2023 European Society of Cardiology guidelines for the management of endocarditis recommend considering a delay of at least four weeks after hemorrhagic stroke in clinically stable patients, while urgent or emergent surgery should be considered in those who are hemodynamically unstable, with the risks and likelihood of meaningful neurologic recovery taken into account [12]. This is consistent with a 2018 systematic review and meta-analysis of 27 observational studies, which found that surgery performed within seven to 14 days of a neurologic event (ischemic or hemorrhagic stroke) was associated with higher perioperative mortality (pooled relative risk 1.74) and greater neurologic exacerbation compared with later surgery [13]. Our patient's hemorrhage was small-volume, and he remained hemodynamically stable without decompensated heart failure, supporting a decision to prioritize neurologic stabilization via aneurysm clipping and management of the postoperative subdural hematoma before proceeding to valve replacement, a sequence consistent with both the guideline framework and the pooled observational evidence [12]. 

Aneurysm clipping, followed by treatment of the postoperative subdural hematoma with MMA embolization, ultimately allowed for the subsequent initiation of anticoagulation and valve replacement. Reassessment by neurology, neurosurgery, and cardiology was required to determine which pathology posed the greatest threat to the patient and, ultimately, to decide the sequence of interventions. This staged approach, with multidisciplinary clearance, struck a balance between preventing progression of the valve disease and mitigating the risk of neurologic hemorrhage, ultimately leading to a favorable patient outcome. This case illustrates the rare but critical scenario in which neurovascular complications directly dictated the timing of subsequent cardiac surgery.

## Conclusions

This case highlights the complex neurologic sequelae of IE, including ischemic stroke, intracranial mycotic aneurysm, and intracranial hemorrhage. The primary clinical lesson of this case is that neurologic complications may directly determine the timing and sequence of cardiac surgery in patients with IE. In this case, multidisciplinary management and neurological stabilization allowed for subsequent cardiac intervention while balancing the risks of intracranial hemorrhage and valvular disease. Successful management required collaboration to address competing neurologic and cardiac risks, allowing treatment of both the neurovascular and cardiac manifestations of *Enterococcus faecalis* IE and resulting in a favorable clinical outcome in a complex case.
